# The Effects of Dental Vulcanite upon Certain Metals

**Published:** 1902-04-15

**Authors:** 


### THE EFFECTS OF DENTAL VULCANITE UPON CERTAIN METALS.

The dental vulcanite contains up to forty percent sulfur. Quite a number of metals possess a great affinity for sulfur, such as copper, silver, steel, etc.; while others, viz., gold, platinum, aluminum, tin, etc., do not share this affinity at all, or in a very mild degree. Those metals which possess a marked affinity for sulfur, can not be utilized in conjunction with vulcanite for the construction of artificial dentures, as the metals will be totally destroyed by the newly-formed sulfur compounds during the process of vulcanization. On the other hand, those metals which form *no* compounds with sulfur do not take a firm hold upon the vulcanite during vulcanization. This statement seems to be paradoxical, but it is, nevertheless, correct. Aluminum-bronze, an alloy consisting of ninety parts copper and ten parts aluminum and much employed by European dentists, is valueless in conjunction with vulcanite; sulfur-compounds of copper are formed rendering the metal valueless. This holds good also for silver and steel. Steel, in the form of piano-wire, is often recommended for strengthening lower plates. By sawing through a plate in which a steel wire is imbeded, one will find the wire in a tube, surrounded by a black deposit. The plate therefore, is not strengthened but rather weakened. If those metals are tinned previous to their imbeding into the rubber, no metal-salts are formed and the desired effect is obtained. All those metals or alloys which form a superficial junction between rubber and metal are recommended for vulcanite work. Gold plate, twelve to eighteen carat fine and dental-alloy, consisting of one part platinum and two parts silver, produce the best union between the metal and the rubber. A good German silver, which should be composed of eight parts copper, three and one-half parts zinc and four parts nickel, will also combine very firmly with vulcanite.—Dr. Polscher, in *Zahntechnische Rundschau*.

In accordance with the above statement we publish herewith the results of a series of experiments made by Dr. Jung to determine the adhesion of certain metals and alloys to vulcanite rubber. This table is taken from Detzner's Work on Prosthetic Dentistry, published by Ash & Sons, Berlin.—Eds. *Dental Era*.

Dr. Jung cut the various metals in pieces about ¼ inch square, on one side a strong hook was soldered while the other polished surface was brought in intimate contact with the vulcanite. After vulcanization, the vulcanite blocks were fastened in a vise and weights adjusted until the metal separated from the vulcanite. The following results were obtained:

*Aluminum-bronze:* rubber detached without weight; metal black and corroded.

*Aluminum:* rubber detached without weight; metal and rubber have polished surfaces.

*Nickel:* rubber detached by 4½ lb. weight; metal and rubber have polished surfaces.

*Nickel-bronze:* rubber detached by 50 lb. weight; metal polished, rubber presents metallic surface.

*German Silver:* rubber detached by 8½ lb. weight; metal polished, rubber presents metallic surface.

*Vitoria Metal:* rubber separated by 60 lb. weight; otherwise same as above.

*Silver:* no attachment of rubber; metal badly corroded and has lost much of its weight.

*Platinum:* no attachment; metal and rubber polished.

*Dental Alloy:* rubber detached by 80 lb. weight; metal polished, rubber presents metallic surface.

*Gold*, *24 carat:* no attachment; metal and rubber polished.

*Gold*, *18 carat:* rubber detached by 140 to 200 lb. weight. Rubber leaves polished surface; gold is tarnished black.

*Gold*, *14-16 carat:* results are the same as with 18 carat gold.

*Gold*, *8 carat:* rubber detached by 45 lb. weight, partly corroded; rubber blackened.

C. Ash & Sons' Orange Rubber was used in these experiments.

The experiments show that the sulfur present in the dental rubber has to form with the metal, up to a certain degree, a new compound which brings about a close adhesion of the two substances. Platinum and high-carat gold, are, therefore, not to be recommended for clasps, etc.; 14 to 18 carat gold or dental alloy will give more satisfactory results.—*Dental Era*.

